# Shock-synthesized quasicrystals

**DOI:** 10.1107/S2052252520005254

**Published:** 2020-04-23

**Authors:** Péter Németh

**Affiliations:** a Institute of Materials and Environmental Chemistry, Research Centre for Natural Sciences, Magyar tudósok körútja 2, Budapest, 1117, Hungary; b Department of Earth and Environmental Sciences, University of Pannonia, Egyetem út 10, Veszprém, 8200, Hungary

**Keywords:** shock synthesis, icosahedral Al_62_Cu_31_Fe_7_, quasicrystals, Khatyrka meteorite

## Abstract

Quasicrystals (QCs) are unique materials, whose crystallographic identity has been controversial for many years. Using recent advances in shock synthesis, Hu *et al.* [(2020), *IUCrJ*, **7**, 434–444] reproduce for the first time the formation conditions of the icosahedral Al_62_Cu_31_Fe_7_ QC (i-phase II) found in the Khatyrka meteorite. The authors demonstrate that shock-recovery experiments provide a novel strategy for preparing and exploring QCs.

Impact-processing of planetary materials and laboratory-shock experiments are of great interest. During these extreme processes a hypervelocity shock wave generates extremely high pressure and high temperature for exceptionally short times (micro- and nano­seconds) and provides favourable conditions for the formation of peculiar structures. In a recent **IUCrJ** article, Hu *et al.* (2020 ▸) report the shock synthesis of an unusual material, the icosahedral Al_62_Cu_31_Fe_7_ quasicrystal (QC), which was previously identified in the Khatyrka meteorite (Bindi *et al.*, 2016 ▸).

QCs are a unique type of material, which are characterized by sharp diffraction features and possess symmetry elements that apparently are inconsistent with the conventional rules of crystallography. In particular, the first Al_86_Mn_14_ alloy QC to be recognized has icosahedral symmetry featuring among other things fivefold rotation axes (Shechtman *et al.*, 1984 ▸). The appearance of these unconventional rotations triggered exciting discussions right from the first report [reviewed in Prodan *et al.* (2017 ▸) and https://paulingblog.wordpress.com/tag/quasicrystals/]. On the one hand, Shechtman and colleagues presented QCs as a new type of crystalline material, in which atoms are repeated according to a quasiperiodic translation (Lifshitz, 2003 ▸). The interpretation of the QCs’ structure was also approached with the so-called Amman tiling, the 3D equivalent of the 2D Penrose tiling (Lord *et al.*, 2000 ▸). On the other hand, Pauling insisted that no crystallographic rules were violated and considered QCs as multiply twinned cubic structures (*e.g.* Pauling, 1985 ▸). Although it turned out that Pauling’s interpretation did not hold, recently Prodan *et al.* (2017 ▸) reported that QCs could in fact be interpreted as multiply twinned structures, considering the unit cell of the primitive golden rhombo­hedra, and demonstrated that ‘tiling’ and ‘multiple twinning’ are fully compatible with each other. However, this interpretation requires the occurrence of equal size twin domains, which is uncommon in nature. In either way, the crystallographic identity of QCs seems to be still debated.

Since their discoveries, over a hundred QCs have been described from syntheses. In contrast, only a few studies report QCs from natural materials and, in fact, all have been associated with Khatyrka meteorite. So far three new QC types have been described from this meteorite: the i-phase I with composition of Al_63_Cu_24_Fe_13_, officially named icosahedrite (Bindi *et al.*, 2009 ▸); the d-phase with composition of Al_71_Ni_24_Fe_5_, referred to as decagonite (Bindi *et al.*, 2015 ▸); and the i-phase II with Al_62_Cu_31_Fe_7_ composition (Bindi *et al.*, 2016 ▸). However, this list perhaps can be extended with additional extraterrestrial phases (personal communication with Luca Bindi).

In either way, to date three natural QCs are known from the Khatyrka meteorite. In meteorite research the mineral assemblage can aid understanding of the formation condition of an associated phase; however, the proper interpretation demands a laboratory-controlled synthesis. Hu *et al.* apply recent advances in the Graded Density Impactor fabrication technique (Kelly *et al.*, 2019 ▸) to shock-synthesize i-phase II. The authors use a special Al–Cu–W disk in an Fe-bearing 304 stainless-steel target chamber and perform two experiments corresponding to an estimated peak shock pressure of 20–30 GPa for 800 ns and 30–35 GPa for 600 ns, respectively. The careful sample characterization, including the state-of-the-art electron backscattered diffraction method, reveals the first experiment (Fig. 1 ▸) resulted in coexisting intermetallic phases of i-phase II, stolperite (β, AlCu) + khatyrkite (θ, Al_2_Cu), which perfectly matches with the assemblage found in Khatyrka meteorite. The second experiment shows the formation of a rather Fe-rich quinary i-phase (Al_68.6_Fe_14.5_Cu_11.2_Cr_4_Ni_1.8_), together with stolperite (β, AlCu) and hollisterite (λ, Al_13_Fe_4_).

A key finding of Hu *et al.* is the shock-synthesized i-phase II, because it represents the first example of a quasicrystal composition discovered in nature prior to being synthesized in the laboratory. Although i-phase I (icosahedrite) together with the d-phase (decagonite) were previously produced by Al-alloy quenching experiments and reported to be thermodynamically stable with a very narrow range of composition (Tsai *et al.*, 1987 ▸), such laboratory conditions were likely inconsistent with those experienced by the Khatyrka meteorite. Furthermore, the previous Al-alloy quenching experiments could not explain the stability of i-phase II and its relationship to i-phase I. Now the authors’ findings provide unique insights into the intricate details of the Al–Cu–Fe phase diagram. In particular, they suggest the thermodynamically stable Al_63_Cu_24_Fe_13_ phase could separate into two disconnected fields under shock pressure above 20 GPa, leading to the co-existence of Fe-rich (i-phase I) and Fe-poor (i-phase II) QCs consistent with the previous findings of Khatyrka meteorite (Bindi *et al.*, 2016 ▸).

QC formation requires controlled syntheses including rapid quenching, during which unusual crystal nucleation and growth can be favoured. The new findings reported by Hu *et al.* demonstrate that shock-recovery experiments provide a novel strategy for preparing and exploring this exciting material group.

## Figures and Tables

**Figure 1:**
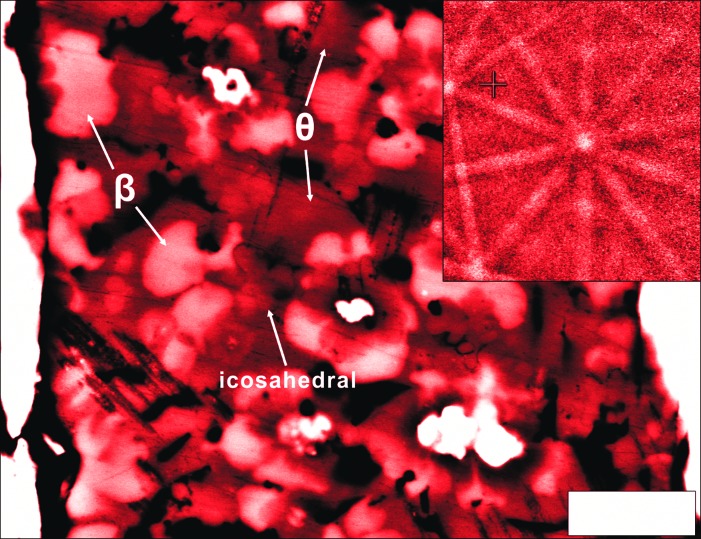
The Al_62_Cu_31_Fe_7_ icosahedral quasicrystal revealing fivefold rotation and its coexisting Cu–Al phase assemblage of stolperite (**β**, AlCu) + khatyrkite (**θ**, Al_2_Cu) from a laboratory-shocked sample. Adapted from Hu *et al.* (2020 ▸).
