# Shock-synthesized quasicrystals. Corrigendum

**DOI:** 10.1107/S2052252521000907

**Published:** 2021-02-06

**Authors:** Péter Németh

**Affiliations:** a Institute of Materials and Environmental Chemistry, Research Centre for Natural Sciences, Magyar tudósok körútja 2, Budapest, 1117, Hungary; b Department of Earth and Environmental Sciences, University of Pannonia, Egyetem út 10, Veszprém, 8200, Hungary

**Keywords:** shock synthesis, icosahedral Al_62_Cu_31_Fe_7_, quasicrystals, Khatyrka meteorite

## Abstract

A statement in the article by Németh [*IUCrJ*, (2020), **7**, 368–369] is corrected.

## Abstract 2

Corrigendum to the article by Németh [*IUCrJ*, (2020), **7**, 368–369].

The statement ‘However, this interpretation requires the occurrence of equal size twin domains, which is uncommon in nature. In either way, the crystallographic identity of QCs seems to be still debated.’ in the Commentary by Németh (2020 ▸) should be replaced by ‘This short Commentary is not intended to make a decision on this matter but notes that there still seems to be a debate about the crystallographic identity of QCs.’
